# IVIg-induced false positive hepatitis B serology in a patient with Guillain-Barré syndrome: a case report

**DOI:** 10.11604/pamj.2025.52.20.49000

**Published:** 2025-09-17

**Authors:** Yasmine Bendimrad, Lina Seffar, Malak Snoussi, Jalila El Bakkouri

**Affiliations:** 1 Faculty of Pharmacy, Mohammed VI University of Sciences and Health, Casablanca, Morocco,; 2 Laboratory Medicine, Cheikh Khalifa International University Hospital, Mohammed VI University of Sciences and Health, Casablanca, Morocco,; 3 Faculty of Medicine, Mohammed VI University of Sciences and Health, Casablanca, Morocco,; 4 Immunopathology-Immunotherapy-Immunomonitoring Laboratory, Mohammed VI University of Sciences and Health, Casablanca, Morocco

**Keywords:** Intravenous immunoglobulin, Guillain-Barré syndrome, false positive, serological interference, case report

## Abstract

We report the case of a 63-year-old Moroccan male with Guillain-Barré syndrome (GBS) who, following treatment with intravenous immunoglobulin (IVIg), developed false-positive anti-HBs and anti-HBc antibodies. This occurred despite negative pre-treatment serology and led to diagnostic confusion. A negative hepatitis B viral DNA PCR confirmed the absence of an active infection. This case highlights a crucial takeaway lesson for clinicians: IVIg can interfere with viral serological assays, and this possibility should be considered to avoid misinterpretation and unnecessary interventions.

## Introduction

Guillain-Barré syndrome (GBS) is an acute inflammatory polyradiculoneuropathy characterized by progressive symmetrical muscle weakness and diminished reflexes [1]. Although its precise pathogenesis remains unclear, it is frequently preceded by bacterial or viral infection, most commonly involving pathogens such as *Campylobacter jejuni*, Cytomegalovirus (CMV), Epstein-Barr virus (EBV), and *Mycoplasma pneumonia* [2,3]. Standard treatment relies on plasmapheresis or immunomodulation with intravenous immunoglobulins (IVIg) [4]. IVIg preparations, derived from pooled plasma of thousands of donors, contain a broad spectrum of polyclonal IgG antibodies, which may lead to passive transfer [5]. This can lead to transient false-positive serological results for various infectious diseases, creating significant diagnostic challenges [6]. We report a case of transient anti-HBs (antibodies to hepatitis B surface antigen) and anti-HBc (antibodies to hepatitis B core antigen) seropositivity following IVIg administration for GBS, despite negative pre-treatment serology and absence of hepatitis B infection. This case highlights the importance of recognizing IVIg-induced serological interference to avoid misdiagnosis and unnecessary interventions.

## Patient and observation

**Patient information:** a 63-year-old Moroccan man with a history of chronic smoking and urinary lithiasis was admitted for acute inflammatory polyradiculoneuropathy consistent with Guillain-Barré syndrome (GBS). He presented with progressive symmetrical limb weakness and areflexia. His medical and family histories were otherwise unremarkable. There was no relevant genetic history.

**Clinical findings:** on admission, neurological examination confirmed symmetrical limb weakness and absence of tendon reflexes, findings consistent with GBS.

**Timeline of current episode:** baseline hepatitis B serology before treatment was negative. He received a standard 5-day course of intravenous immunoglobulin (IVIg), which resulted in neurological improvement and enabled his initial discharge. However, on day 15 after admission, he was readmitted with diffuse purpuric lesions, abdominal pain, and acute kidney injury. By day 20, repeat hepatitis B serology showed seroconversion with positive anti-HBs and anti-HBc antibodies. With adequate supportive care and treatment, his condition improved progressively. A chronological summary of the patient's clinical course is presented in Table 1.

**Table 1:** timeline of clinical events, treatments, and laboratory findings in the reported patient

Timepoint	Clinical events and findings
Admission	Hospitalized for GBS; neurological exam confirmed symmetrical weakness and areflexia.
Baseline (pre-IVIg)	Hepatitis B serology: negative for HBsAg, anti-HBc, anti-HBs.
Day 1-5	Standard 5-day IVIg therapy (0.4 g/kg/day).
Post-IVIg	Neurological improvement allowed discharge.
Day 15 post-admission	Readmission with diffuse purpura, abdominal pain, and acute kidney injury.
Day 20 post-admission	Liver workup: hepatitis B seroconversion (positive anti-HBs and anti-HBc).
Later	Progressive clinical improvement under supportive care.

**Diagnostic assessment:** cerebrospinal fluid analysis showed albuminocytologic dissociation, with elevated protein concentration (216 mg/dL; normal 15-45 mg/dL) and normal cell count (<5 cells/mL), supporting GBS diagnosis. Skin biopsy during the second admission confirmed leukocytoclastic vasculitis with IgA deposits, consistent with Henoch-Schönlein purpura. Hepatitis B serology results before and after IVIg administration are summarized in Table 2. HBV DNA PCR was negative, excluding active infection.

**Table 2:** evolution of hepatitis B serological markers before and after IVIg administration

Investigation	Reference intervals (units)	Before IVIg Interpretation	Value	Post-IVIg Interpretation	Value
HbsAg	< 1 (S/CO)	Negative	0.20	Negative	0.21
Anti-HBs	< 10 (mIU/mL)	Negative	0.77	Positive	41.22
Anti-HBc	< 1 (S/CO)	Negative	0.09	Positive	1.53

HBsAg: Hepatitis B surface antigen; Anti-HBs: Antibodies to hepatitis B surface antigen; Anti-HBc: Total antibodies to hepatitis B core antigen; IVIg: Intravenous immunoglobulin, S/CO: Signal to Cut-Off ratio

**Diagnosis:** Guillain-Barré syndrome treated with IVIg, complicated by Henoch-Schönlein purpura confirmed by skin biopsy, and transient hepatitis B seropositivity secondary to passive antibody transfer.

**Therapeutic interventions:** initial treatment consisted of a standard 5-day course of IVIg therapy (0.4 g/kg/day). During the second admission, management included corticosteroids, antibiotics, intravenous albumin infusions, and diuretics.

**Follow-up and outcome of interventions:** the transient hepatitis B antibody positivity was attributed to passive transfer from prior IVIg administration, consistent with the known pharmacokinetics and antibody half-life. Repeat serology performed during follow-up confirmed the gradual decline of anti-HBs and anti-HBc levels without any evidence of active infection. The Henoch-Schönlein purpura improved with corticosteroid therapy and supportive care, with complete resolution of skin lesions and abdominal pain. Renal function progressively normalized, and the patient's neurological status also improved, allowing discharge with full clinical recovery.

**Patient perspective:** the patient expressed relief upon learning that the abnormal hepatitis B results were not due to an actual infection and appreciated the clear explanation regarding IVIg-related serological interference.

**Informed consent:** written informed consent was obtained from the patient for publication of this case report and accompanying data.

## Discussion

This case illustrates a well-recognized but often overlooked diagnostic challenge in patients receiving intravenous immunoglobulin (IVIg): passive transfer of antibodies, resulting in transient false-positive serological results. Our patient, seronegative for hepatitis B virus (HBV) markers, before therapy, developed transient anti-HBs and anti-HBc positivity after IVIg administration, with no evidence of active infection.

IVIg preparations, produced from pooled plasma of thousands of donors, contain a broad spectrum of polyclonal immunoglobulins [7]. These may include antibodies directed against HBV and other infectious agents, which can be transferred passively to recipients. Such a transfer can create misleading serological profiles, particularly when testing is performed soon after infusion.

False positive serologies following IVIg have been documented for several pathogens, including HBV, hepatitis C virus (HCV), and human immunodeficiency virus (HIV) [8,9]. The half-life of passively transferred antibodies is usually 3 to 4 weeks, aligning with the transient pattern observed in our case [10]. In the context of HBV, differentiating between true infection and passive antibody transfer is essential, especially in patients who are immunocompromised or candidates for immunosuppressive therapy. A negative hepatitis B surface antigen (HBsAg) and undetectable HBV DNA by PCR, combined with the clinical context, strongly support the diagnosis of passive transfer rather than active infection.

From a clinical perspective, awareness of this phenomenon is crucial to avoid unnecessary investigations, misdiagnosis or inappropriate treatment. When possible, baseline serologies should be obtained before IVIg therapy, and repeat testing after 4-6 weeks can help confirm antibody clearance.

A key strength of this report is the clear documentation of the patient's initial seronegativity and the use of HBV DNA PCR to definitively rule out a true infection. A limitation, however, is the absence of serial quantitative antibody titers beyond day 20, which would have provided a more detailed kinetic profile of the antibody decline. Nevertheless, the clinical context, initial seronegativity, negative HBV DNA PCR, and the spontaneous resolution of the clinical picture strongly support the interpretation of passive antibody transfer.

## Conclusion

This case underscores the importance of recognizing IVIg-induced serological interference, particularly in hepatitis B testing. Passive antibody transfer can produce transient false positive results, potentially leading to unnecessary procedures or treatment. Obtaining baseline serology before IVIg and interpreting post-treatment results cautiously can prevent misinterpretation and improve patient safety.
